# CETP inhibitors and cardiovascular disease: Time to think again

**DOI:** 10.12688/f1000research.4396.1

**Published:** 2014-06-10

**Authors:** Norman E Miller

**Affiliations:** 1 Magdalen College, University of Oxford, Oxford, OX1 4AU, UK

## Abstract

Inhibition of cholesteryl ester transfer protein (CETP) lowers plasma low-density lipoprotein cholesterol concentration and raises high-density lipoprotein (HDL) cholesterol, suggesting it might prevent cardiovascular disease (CVD). From the outset, however, the concept has been controversial owing to uncertainty about its effects on HDL function and reverse cholesterol transport (RCT). Although there has long been good evidence that CETP inhibition reduces atherosclerosis in rabbits, the first information on CETP as a CVD risk factor in a prospectively followed cohort was not published until after the first Phase 3 trial of a CETP inhibitor had begun. The worrying finding that CVD incidence was related inversely to plasma CETP has since been reproduced in each of five further prospective cohort studies. Similar results were obtained in subjects on or off statin therapy, for first and second CVD events, and for mortality as well as CVD morbidity. Additionally, two recent studies have found alleles of the *CETP *gene that lower hepatic CETP secretion to be associated with an increased risk of myocardial infarction. Meanwhile, *CETP* gene transfer in mice was found to increase RCT from peripheral macrophages *in vivo*, and human plasma with high CETP activity was shown to have a greater capacity to remove cholesterol from cultured cells than plasma with low activity. This mounting evidence for a protective function of CETP has been given remarkably little attention, and indeed was not mentioned in several recent reviews.  It appears to show that CETP inhibition does not test the HDL hypothesis as originally hoped, and raises a pressing ethical issue regarding two Phase 3 trials of inhibitors, involving more than forty thousand subjects, which are currently in progress. As the weight of evidence now clearly supports an adverse effect of CETP inhibition on CVD, an urgent review is needed to determine if these trials should be discontinued.

## Background

Cholesteryl ester transfer protein (CETP) is a hydrophobic glycoprotein in plasma that catalyzes the transfer of neutral lipids between plasma lipoproteins ^1^. The notion that inhibition of CETP activity might prevent coronary heart disease (CHD) was based on the knowledge that it both reduces plasma low-density lipoprotein (LDL) cholesterol concentration, and raises high-density lipoprotein (HDL) cholesterol. While there is abundant evidence that reduction of LDL cholesterol is likely to be beneficial, the effect of CETP inhibition on the function of HDL in reverse cholesterol transport from tissues (RCT) has been uncertain.

In 1996 Fielding and Havel ^2^ argued against a hasty commitment to CETP inhibitors, drawing attention to evidence that CETP participates in the remodelling of the cholesteryl ester-rich α-HDLs that generates the small lipid-poor preβ-HDLs that are the primary acceptors of cholesterol via the ABCA1 transporters in cell membranes ^3^. The rise in HDL cholesterol might be misleading, they argued, and reflect only retention of cholesteryl esters in the particles, while the uptake of cholesterol from arterial cells is diminished. Nevertheless, encouraged by reports that *CETP* gene transfer induced atherosclerosis in mice ^4^ and that CETP inhibition prevented atherosclerosis in rabbits ^5– 7^, drug discovery programmes made rapid progress.

The case for inhibition was weakened when later studies of CETP transgenic mice contradicted the earlier findings ^8, 9^, and the incidence of CHD was not found to be significantly reduced in familial CETP deficiency ^10^. Studies of the relation of CHD to single nucleotide polymorphisms (SNPs) of the *CETP* gene yielded disparate outcomes, which were not resolved by meta-analyses ^11– 13^. When Dullaart *et al.* ^13^ meta-analyzed data on the *Taq1B* SNP (rs708272) from population-based and high-risk groups separately, the odds ratio for cardiovascular disease (CVD) in homozygotes for the *B2* allele (who have low CETP activity) was 0.84 in the high-risk subjects, but 1.45 in the population-based samples. This suggested that low CETP activity actually increases CVD risk, and that its seeming protective effect in some studies may have resulted from selection towards a lower frequency of the *B2* allele in high-risk groups.

The dual uncertainties over the effect of CETP inhibition on HDL function and whether it is more likely to reduce or increase CVD in humans were unresolved when the first Phase 3 study (ILLUMINATE; NCT00134264) of a member of this class of drugs (torcetrapib) was started in July 2004 ^14^. In June 2007, the trial was terminated after it had become clear that the treatment had increased the incidence of the primary CVD endpoint. The authors concluded that this was probably owing to an unanticipated rise in blood pressure, but three findings challenged this interpretation. CHD mortality was related inversely to increment in blood pressure; the incidence of stroke was not raised by treatment; and non-cardiovascular mortality was increased. A Phase 3 trial of dalcetrapib (Dal-OUTCOMES; NCT00658515), a less potent inhibitor with little or no effect on blood pressure, was started in April 2008. In November 2012, this study was also terminated when it became evident that the outcome was not going to be positive ^15^.

In the same year that ILLUMINATE enrolled its first participant, the first prospective data on plasma CETP as a risk factor for clinical CVD events were also published. Boekholdt *et al.* ^16^ had found that CETP concentration, with which CETP activity is linearly related ^17^, did not differ significantly between 1,400 controls and 735 subjects who had developed myocardial infarction (MI) during six years of follow-up, although a positive association was seen on *post hoc* analysis in subjects with plasma triglycerides exceeding the median of 1.7 mmol/l.

## Recent findings on plasma CETP as a CVD risk factor

The first prospective observational cohort study of plasma CETP activity or concentration as a risk factor for clinical CVD events did not appear until July 2006, two years after ILLUMINATE had started. Since then five further similar studies have been published. All six studies found CVD incidence to be related *inversely* to plasma CETP ^18– 23^. The designs and results of these studies are summarised in Table 1. Cohorts ranged from 1,002 to 3,256 subjects, and follow-up periods from two to 15 years (weighted average, 7.6 years). One study followed men and women separately ^23^. Three were of first CVD events in healthy subjects ^18, 19, 23^; two were of second events in subjects with an existing history of CHD ^20, 21^; and in one study primary and secondary events were pooled ^22^. In two studies that looked at mortality in addition to CVD morbidity ^21, 22^ this was also negatively associated with CETP. Results in subjects taking pravastatin or atorvastatin ^18, 20^ mirrored those in other subjects. The suggestion in the earlier case-control study ^16^ that subjects with raised triglycerides might differ from others was not confirmed.

**Table 1.** Prospective observational cohort studies of CVD risk and plasma CETP concentration or activity.

Study	Design	Results
Marschang 2006 ^18^	1002 subjects (mean age, 65 yr; 44% female) free of CVD and taking pravastatin were followed for two years, during which 100 suffered a CVD event.	Significantly more CVD events in the bottom quartile of CETP conc than in the top quartile (OR 3.2, P=0.001). The association remained significant (P=0.001) after adjustment for age, sex, lipids and other risk factors. Similar result observed for cardiac events alone (P=0.009).
Vasan 2009 ^19^ Framingham Heart Study	1978 subjects free of CVD (mean age, 51 yr, 54% female) were followed for 15 years, during which 320 suffered a CVD event.	CVD incidence was negatively associated with CETP activity (P=0.004) after adjustment for age, sex and standard risk factors. CETP activity ≥ median was associated with a risk reduction of 30%.
Khera 2010 ^20^ PROVE IT-TIMI 22 Study	3218 subjects (mean age, 58 yr; 21% female) with clinical CHD taking either atorvastatin or pravastatin were followed for two years, during which 150 suffered a recurrent AMI or CHD death.	CETP conc was negatively associated with CHD risk in unadjusted data (HR per SD increase 0.77, P=0.005) and after adjustment for age, sex and other risk factors (0.81, P=0.027). In subjects whose LDL was below the median, CETP conc above the median was associated with a HR of 0.52 (P=0.02).
Duwensee 2010 ^21^ KAROLA Study	1132 subjects with clinical CHD (mean age 59 yr, 15% female, 18% diabetics) were followed for 8 yr, during which there were 150 cases of fatal or non-fatal CVD, and 119 deaths.	With or without adjustment for sex, age and standard risk factors, HR for a new CVD event was negatively associated with CETP conc. Relative to subjects with CETP above the median, those below it had a HR for CVD of 1.84 (P<0.02), and for total mortality of 1.57 (P=0.04).
Ritsch 2010 ^22^ LURIC Study	3256 subjects referred for coronary angiography (mean age 62 yr, 30% female), of whom 2560 had verified CAD, were followed for a mean of eight years, during which there were 754 total deaths, and 474 deaths from CVD.	CETP conc was lower in patients with CAD than in those without (P=0.002). Relative to the top CETP quartile, age- and sex-adjusted HR for CVD death in the bottom quartile was 1.38 (P=0.02), and that for total mortality was 1.37 (P=0.004). HRs were unaffected by adjustment for other risk factors.
Robins 2013 ^23^ Framingham Offspring Study	2679 subjects (mean age, 58 yr, 56% female) free of CVD were followed for a mean of 10.4 yr, during which 187 suffered a CVD event.	In men CVD risk was associated negatively with CETP activity and positively with PLTP activity. After adjustment for PLTP and other risk factors, CVD remained negatively associated with CETP activity (HR 0.64, P=0.026). No significant associations in women.

Studies limited to coronary angiography without clinical endpoints are not included. AMI, acute myocardial infarction; BMI, body mass index; CAD, coronary artery disease; CHD, coronary heart disease; CVD, cardiovascular disease; HR, hazard ratio; OR, odds ratio; PLTP, phospholipid transfer protein.

## Recent genome-wide analyses of *CETP* alleles and CVD

The *CETP* gene has been mapped to locus 16q21. It spans about 25 kb, and consists of 16 exons and 15 introns. In the absence of a clear picture from candidate gene studies of the association of SNPs with CVD, two genome-wide analyses have recently been published, whose results appeared to conflict with those of the observational epidemiology. In a study of more than 350,000 SNPs in 18,245 women followed for 10 years, Ridker *et al.* ^24^ observed that three SNPs in or around the *CETP* gene (rs708272, rs4329913, rs7202364) were associated with increased HDL cholesterol and a reduced incidence of MI. A subsequent Mendelian randomization analysis found a single SNP of *CETP* (rs3764261) to be associated with raised HDL cholesterol and an apparent four per cent reduction in the incidence of MI ^25^.

## Reconciling the observational epidemiology and genome-wide analyses

The reliability of prospective observational epidemiology for the identification of causal effects in complex diseases has been in the spotlight of late, after some results were not confirmed in randomized clinical trials. Could the results of the recent observational studies of CETP be another instance of confounding or reverse causation? Confounding seems unlikely as multivariate analyses found the relation between CVD events and CETP concentration or activity to be independent of age, gender, hypertension, body mass index, plasma triglycerides, adiponectin, diabetes, and smoking habit ^18– 23^. Reverse causation due to a reduction of plasma CETP in response to vascular inflammation also seems improbable, as the association persisted after adjustment for plasma homocysteine, interleukin-6 and C-reactive protein concentrations ^22, 23^.

As discussed by several authors ^26– 28^, genome-wide analyses are also not without their limitations, and several aspects of the two studies warrant consideration. One is that there appears to have been no concordance between them in the alleles found to be associated with MI. Second, as data on plasma CETP were not available to either study, the relations with disease could have been owing to linkage with other genes that affect HDL and MI through independent mechanisms. The strongest association in the first study ^24^ was with rs708272, the *Taq1B* SNP of *CETP*. This intronic polymorphism has no direct affect on CETP activity. Furthermore, the allele associated with low incidence of MI has also been found to be associated with a low prevalence of metabolic syndrome ^29^, a potential confounder being itself a strong risk factor for CVD. The other two alleles were remote from the *CETP* gene, being in *SLC12A3* and *NUP93,* respectively a solute transporter gene and the gene for a nucleoporin. The relation of MI to rs3764261 described in the more recent genome-wide study was adjusted for age and sex, but not for other potential confounders ^25^. Furthermore, the result has since been contradicted by a meta-analysis of data from 16,570 subjects ^30^, which found the *T* allele of the same SNP to be associated with reduced effectiveness of statins in preventing MI.

Papp *et al.* ^31^ have recently addressed the issue of genetically determined low CETP activity by using mRNA allelic expression and splice isoform assays to identify genetic variants that affect plasma CETP concentration, and then examining their relation to incident MI. In studies of 94 human livers, a common alternatively spliced isoform lacking exon 9 prevented CETP secretion in a dominant-negative manner. Increased formation of this isoform was exclusively associated with two polymorphisms in high linkage disequilibrium: one in exon 9 of *CETP* ( *rs5883-C>T*), which alters an exonic splicing enhancer sequence, and another in intron 8 ( *rs9930761-T>C*), which changes a splicing branch point nucleotide. In the INVEST-GENES prospectively followed cohort, it was found that rs5883T/rs9930761C were associated with high incidence rates of MI and stroke (P = 0.005) despite raised HDL cholesterol, strongly reinforcing the observational epidemiology.

## Recent studies of CETP, HDL function and reverse cholesterol transport

While the epidemiologic landscape has thus evolved, laboratory research has strengthened the evidence that CETP plays an important role in RCT. Tanigawa *et al.* ^32^ found that hepatic *CETP* gene transfer in mice stimulated the transport of cholesterol from peritoneal macrophages to the liver, followed by its elimination as bile acids. Tchoua *et al.* ^33^ independently confirmed this result, and showed that the effect was blocked when the animals were given torcetrapib. There is no accepted method for quantifying reverse cholesterol transport *in vivo* in humans, but three groups have recently reported that human plasma with high CETP activity had a greater capacity to promote cholesterol efflux from cultured cells than plasma with low activity ^34– 36^. Villard *et al.* ^34^ showed further that addition of purified CETP increased both the preβ-HDL concentration in normal human plasma and its capacity to remove cholesterol from cultured cells, reproducing an earlier result obtained with plasma from a subject with familial CETP deficiency ^37^. Thus, the confusion over the contribution of CETP to RCT appears to have been resolved, and the concerns expressed by Fielding and Havel almost 20 years ago substantiated.

## Perspective

The history of the hypothesis that CETP inhibition will prevent atherosclerosis can be summarised thus. At the outset, our understanding of HDL biochemistry did not permit any predictions of its effect on RCT, but was sufficient to tell us that it might go either way. In the absence of information on the relation of CVD risk to CETP activity in humans, enthusiasm for the concept was fuelled by positive results in cholesterol-fed rabbits, which seemed to confirm that a rise in HDL cholesterol is a dependable biomarker of benefit. However, the first prospective cohort study of CETP as a CVD risk factor challenged this assumption. Since then, five further prospective observational studies have left no doubt that in populations CVD risk is related inversely to CETP activity. Confounding and reverse causation seem unlikely explanations. Although two genome-wide analyses appeared to have produced contrary evidence, for the reasons discussed they have not refuted the observational data. On the other hand, the latter have been reinforced by reports that subjects with functional *CETP* alleles that lower CETP secretion have an increased risk of MI. Thus, the weight of evidence has now shifted to the likelihood that CETP inhibition will have an adverse effect on CVD outcomes, not the beneficial effect that was hoped for. Recent laboratory studies on the impact of CETP activity on the cholesterol transport function of HDL have been consistent with this interpretation.

This interpretation does not conflict with the anti-atherogenic effect of CETP inhibition in rabbits. Apart from the obvious possibility of a species specific difference in cholesteryl ester dynamics, Shimoji *et al.* ^38^ reported that dalcetrapib increases the synthesis rate of the major HDL protein (apo AI) in rabbits by 44 per cent, an effect that on its own would be expected to substantially reduce atherosclerosis ^39^. By contrast, inhibition of CETP with torcetrapib had no effect on apo AI synthesis in humans ^40^. It is also worth noting that probucol, which increases CETP activity ^41^, also prevents atherosclerosis in rabbits despite lowering apo AI synthesis rate ^41, 42^.

Although the body of disquieting data has been growing for several years, there has been surprisingly little public discussion of the issue. The paper describing the outcome of ILLUMINATE ^14^ made no reference to the results of Marschang *et al.* ^18^ published the year before. Likewise, the report on Dal-OUTCOMES ^15^ made no mention of any one of the six observational cohort studies listed in Table 1, all of which were already in print. The same is true of an article investigating the harm caused by torcetrapib in ILLUMINATE ^43^, and of several recent review articles ^44– 48^.

## Implications

These recent developments have significant implications. First, they are consistent with other evidence that plasma HDL cholesterol concentration is not a reliable marker of the efficiency of RCT ^49, 50^. Second, they show that clinical trials of CETP inhibitors do not test the HDL hypothesis in the manner originally envisaged. Third, they raise a pressing issue in the context of two Phase 3 studies of second generation CETP inhibitors currently in progress. ACCELERATE (NCT01687998) ^51^, which began in 2012 and is expected to finish in 2016, has enrolled about 12,000 patients with high-risk CVD to assess the efficacy of evacetrapib ^52^ in preventing CVD events. REVEAL (NCT01252953) ^53^, commenced in 2011 and expected to be completed in 2017, has enrolled 30,624 patients for a similar study of anacetrapib ^54^. In both studies, the patients in each arm are being given a statin to control LDL concentration prior to randomization.

Anacetrapib is the most potent CETP inhibitor to date, and was found in DEFINE (NCT00685776) ^54^ to lower LDL cholesterol by 50 per cent compared with the 25 per cent achieved with torcetrapib ^14^. It is theoretically possible that this greater impact on LDL will override any adverse effect on HDL function, but it is equally possible that its greater impact on HDL cholesterol (140 per cent increase compared with 70 per cent) reflects such an extreme disturbance of HDL metabolism that its consequences will predominate. Neither the prospective epidemiology nor studies of familial CETP deficiency have provided evidence of a fall in CVD risk at extremely low activities. Although DEFINE recorded no increase in CVD in patients given anacetrapib, the authors noted that the study was too small to provide reliable information on clinical events ^54^.

## Conclusion

Given that the tide of evidence has turned so strongly against CETP inhibition in recent years, the question must be asked of whether it is now ethical to continue with the two Phase 3 trials in progress. A clinical trial is considered to be ethical only if it has a sound scientific basis and a favourable risk-benefit balance ^55^. The two trials in question no longer satisfy either requirement, as there is clearly a strong possibility that the drugs will have exactly the opposite effect on CVD to that intended. Some might argue that there is no cause for concern, as morbidity and mortality are being regularly reviewed by data monitoring committees. However, such committees can intervene only when pre-specified statistical criteria have been met, by which time many participants may have suffered harm.

Carrying on and hoping for the best is not an acceptable option. An independent review is urgently needed to determine if the trials should be discontinued.

## References

[1] Fielding CJ Fielding PE: Molecular physiology of reverse cholesterol transport. *J Lipid Res.* 1995;36(2):211–28 7751809

[2] Fielding CJ Havel RJ: Cholesteryl ester transfer protein: friend or foe? *J Clin Invest.* 1996;97(12):2687–8 10.1172/JCI118719 8675675 PMC507357

[3] Hennessy LK Kunitake ST Kane JP: Apolipoprotein A-I-containing lipoproteins, with or without apolipoprotein A-II, as progenitors of pre-beta high density lipoprotein particles. *Biochemistry.* 1993;32(22):5759–65 10.1021/bi00073a006 8504094

[4] Marotti KR Castle CK Boyle TP: Severe atherosclerosis in transgenic mice expressing simian cholesteryl ester transfer protein. *Nature.* 1993;364(6432):73–5 10.1038/364073a0 8316302

[5] Okamoto H Yonemori F Wakitani K: A cholesteryl ester transfer protein inhibitor attenuates atherosclerosis in rabbits. *Nature.* 2000;406(6792):203–7 10.1038/35018119 10910363

[6] Sugano O Makino N Sawada S: Effect of antisense oligonucleotides against cholesteryl transfer protein on the development of atherosclerosis in cholesterol-fed rabbits. *J Biol Chem.* 1998;273(9):5033–6 10.1074/jbc.273.9.5033 9478952

[7] Ritterhaus CW Miller DP Thomas LJ: Vaccine-induced antibodies inhibit CETP activity *in vivo* and reduce aortic lesions in a rabbit model of atherosclerosis. *Arterioscler Thromb Vasc Biol.* 2000;20(9):2106–12 10.1161/01.ATV.20.9.2106 10978256

[8] Hayek T Masucci-Magoulas L Jiang X: Decreased early atherosclerotic lesions in hypertriglyceridemic mice expressing cholesteryl ester transfer protein transgene. *J Clin Invest.* 1995;96(4):2071–4 10.1172/JCI118255 7560101 PMC185846

[9] Föger B Chase M Amar MJ: Cholesteryl ester transfer protein corrects dysfunctional high density lipoproteins and reduces aortic atherosclerosis in lecithin cholesterol acyltransferase transgenic mice. *J Biol Chem.* 1999;274(52):36912–20 10.1074/jbc.274.52.36912 10601244

[10] Curb JD Abbott RD Rodriguez BL: A prospective study of HDL-C and cholesteryl ester transfer protein gene mutations and the risk of coronary heart disease in the elderly. *J Lipid Res.* 2004;45(5):948–53 10.1194/jlr.M300520-JLR200 14967821

[11] Thompson A Di Angelantonio E Sarwar N: Association of cholesteryl ester transfer protein genotypes with CETP mass and activity, lipid levels, and coronary risk. *JAMA.* 2008;299(23):2777–88 10.1001/jama.299.23.2777 18560005

[12] Li YY Wu XY Xu J: Apo A5 -1131T/C, FgB -455G/A, -148C/T, and CETP TaqIB gene polymorphisms and coronary artery disease in the Chinese population: a meta-analysis of 15,055 subjects. *Mol Biol Rep.* 2013;40(2):1997–2014 10.1007/s11033-012-2257-9 23129316

[13] Dullaart RP Sluiter WJ: Common variation in the CETP gene and the implications for cardiovascular disease and its treatment: an updated analysis. *Pharmacogenomics.* 2008;9(6):747–63 10.2217/14622416.9.6.747 18518852

[14] Barter PJ Caulfield M Eriksson M: Effects of torcetrapib in patients at high risk for coronary events. *N Engl J Med.* 2007;357(21):2109–22 10.1056/NEJMoa0706628 17984165

[15] Schwartz GG Olsson AG Abt M: Effects of dalcetrapib in patients with a recent acute coronary syndrome. *N Engl J Med.* 2012;367(22):2089–99 10.1056/NEJMoa1206797 23126252

[16] Boekholdt SM Kuivenhoven JA Wareham NJ: Plasma levels of cholesteryl ester transfer protein and the risk of future coronary artery disease in apparently healthy men and women: the Prospective EPIC (European Prospective Investigation into Cancer and Nutrition)–Norfolk population study. *Circulation.* 2004;110(11):1418–23 10.1161/01.CIR.0000141730.65972.95 15337694

[17] Ritsch A Auer B Foger B: Polyclonal antibody-based immunoradiometric assay for quantification of cholesteryl ester transfer protein. *J Lipid Res.* 1993;34(4):673–79 8496672

[18] Marschang P Sandhofer A Ritsch A: Plasma cholesteryl ester transfer protein concentrations predict cardiovascular events in patients with coronary artery disease treated with pravastatin. *J Intern Med.* 2006;260(2):151–9 10.1111/j.1365-2796.2006.01674.x 16882279

[19] Vasan RS Pencina MJ Robins SJ: Association of circulating cholesteryl ester transfer protein activity with incidence of cardiovascular disease in the community. *Circulation.* 2009;120(24):2414–20 10.1161/CIRCULATIONAHA.109.872705 19948972 PMC2818786

[20] Khera AV Wolfe ML Cannon CP: On-statin cholesteryl ester transfer protein mass and risk of recurrent coronary events (from the pravastatin or atorvastatin evaluation and infection therapy–thrombolysis in myocardial infarction 22 [PROVE IT-TIMI 22] study). *Am J Cardiol.* 2010;106(4):451–6 10.1016/j.amjcard.2010.03.057 20691300

[21] Duwensee K Breitling LP Tancevski I: Cholesteryl ester transfer protein in patients with coronary heart disease. *Eur J Clin Invest.* 2010;40(7):616–22 10.1111/j.1365-2362.2010.02313.x 20497463

[22] Ritsch A Scharnagl H Eller P: Cholesteryl ester transfer protein and mortality in patients undergoing coronary angiography: the Ludwigshafen Risk and Cardiovascular Health study. *Circulation.* 2010;121(3):366–74 10.1161/CIRCULATIONAHA.109.875013 20065167 PMC2964596

[23] Robins SJ Lyass A Brocia RW: Plasma lipid transfer proteins and cardiovascular disease. The Framingham Heart Study. *Atherosclerosis.* 2013;228(1):230–6 10.1016/j.atherosclerosis.2013.01.046 23477743 PMC3692011

[24] Ridker PM Paré G Parker AN: Polymorphism of the *CETP* gene region, HDL cholesterol, and risk of future myocardial infarction: Genomewide analysis among 18 245 healthy women from the Women’s Genome Health Study. *Circ Cardiovasc Genet.* 2009;2(1):26–33 10.1161/CIRCGENETICS.108.817304 20031564 PMC2729193

[25] Voight BF Peloso GM Ortho-Melander M: Plasma HDL cholesterol and risk of myocardial infarction: a mendelian randomisation study. *Lancet.* 2012;380(9841):572–80 10.1016/S0140-6736(12)60312-2 22607825 PMC3419820

[26] Smith GD Ebrahim S: Mendelian randomization: prospects, potentials, and limitations. *Int J Epidemiol.* 2004;33(1):30–42 10.1093/ije/dyh132 15075143

[27] Sleiman PM Grant SF: Mendelian randomization in the era of genomewide association studies. *Clin Chem.* 2010;56(5):5723–8 10.1373/clinchem.2009.141564 20224045

[28] Barsh GS Copenhaver GP Gibson G: Guidelines for genome-wide association studies. *PLoS Genet.* 2012;8(7):e1002812 10.1371/journal.pgen.1002812 22792080 PMC3390399

[29] Sandhofer A Tatarczyk T Laimer M: The *Taq1B*-variant in the cholesteryl ester-transfer protein gene and the risk of metabolic syndrome. *Obesity (Silver Spring).* 2008;16(4):919–22 10.1038/oby.2007.130 18239576

[30] Leusink M Onland-Moret NC Asselbergs FW: Cholesteryl ester transfer protein polymorphisms, statin use and their impact on cholesterol levels and cardiovascular events. *Clin Pharmacol Ther.* 2014;95(3):314–20 10.1038/clpt.2013.194 24080640

[31] Papp AC Pinsonneault JK Wang W: Cholesteryl Ester Transfer Protein (CETP) polymorphisms affect mRNA splicing, HDL levels, and sex-dependent cardiovascular risk. *PLoS One.* 2012;7(3):e31930 10.1371/journal.pone.0031930 22403620 PMC3293889

[32] Tanigawa H Billheimer JT Tohyama J: Expression of cholesteryl ester transfer protein in mice promotes macrophage reverse cholesterol transport. *Circulation.* 2007;116(11):1267–73 10.1161/CIRCULATIONAHA.107.704254 17709636

[33] Tchoua U D’Souza W Mukhamedova N: The effect of cholesteryl ester transfer protein overexpression and inhibition on reverse cholesterol transport. *Cardiovasc Res.* 2008;77(4):732–9 10.1093/cvr/cvm087 18056760

[34] Villard EF El Khoury P Duchene E: Elevated CETP activity improves plasma cholesterol efflux capacity from human macrophages in women. *Arterioscler Thromb Vasc Biol.* 2013;32(10):2341–9 10.1161/ATVBAHA.112.252841 22904275

[35] Borggreve SE de Vries R Dallinga-Thie GM: The ability of plasma to stimulate fibroblast cholesterol efflux is associated with the -629C-->A cholesteryl ester transfer protein promoter polymorphism: role of lecithin: cholesterol acyltransferase activity. *Biochim Biophys Acta.* 2008;1781(1–2):10–5 10.1016/j.bbalip.2007.10.010 18178167

[36] Scharnag lH Heuschneider C Sailer S: Decreased cholesterol efflux capacity in patients with low cholesteryl ester transfer protein plasma levels. *Eur J Clin Invest.* 2014;44(4):395–401 10.1111/eci.12248 24467215

[37] Yamashita S Ishigami M Arai T: Very high density lipoproteins induced by plasma cholesteryl ester transfer protein (CETP) have a potent antiatherogenic function. *Ann N Y Acad Sci.* 1995;748:606–8 10.1111/j.1749-6632.1994.tb17372.x 7695214

[38] Shimoji E Zhang B Fan P: Inhibition of cholesteryl ester transfer protein increases serum apolipoprotein (apo) A-I levels by increasing the synthesis of apo A-I in rabbits. *Atherosclerosis.* 2004;172(2):247–57 10.1016/j.atherosclerosis.2003.09.029 15019534

[39] Duverger N Kruth H Emmanuel F: Inhibition of atherosclerosis development in cholesterol-fed human apolipoprotein A-I-transgenic rabbits. *Circulation.* 1996;15(4):94:713–7 10.1161/01.CIR.94.4.713 8772693

[40] Brousseau ME Diffenderfer MR Millar JS: Effects of cholesteryl ester transfer protein inhibition on high-density lipoprotein subspecies, apolipoprotein A-I metabolism, and fecal sterol excretion. *Arterioscler Thromb Vasc Biol.* 2005;25(5):1057–64 10.1161/01.ATV.0000161928.16334.dd 15761191 PMC3229922

[41] Oshima R Ikeda T Watanabe K: Probucol treatment attenuates the aortic atherosclerosis in Watanabe heritable hyperlipidemic rabbits. *Atherosclerosis.* 1998;137(1):13–22 10.1016/S0021-9150(97)00243-8 9568732

[42] Ying H Saku K Harada R: Putative mechanisms of action of probucol on high-density lipoprotein apolipoprotein A-I and its isoproteins kinetics in rabbits. *Biochim Biophys Acta.* 1990;1047(3):247–54 10.1016/0005-2760(90)90523-Z 2123720

[43] Barter PJ Rye KA Beltangady MC: Relationship between atorvastatin dose and the harm caused by torcetrapib. *J Lipid Res.* 2012;53(11):2436–42 10.1194/jlr.P026328 22941786 PMC3466012

[44] Barter PJ Rye KA: Cholesteryl ester transfer protein inhibition as a strategy to reduce cardiovascular risk. *J Lipid Res.* 2012;53(9):1755–66 10.1194/jlr.R024075 22550134 PMC3413218

[45] Durrington PN: Cholesteryl ester transfer protein (CETP) inhibitors. *Br J Cardiol.* 2012;19:126–33 10.5837/bjc.2012.024

[46] Hewing B Fisher EA: Rationale for cholesteryl ester transfer protein inhibitors. *Curr Opin Lipidol.* 2012;23(4):372–6 10.1097/MOL.0b013e328353ef1d 22517614 PMC3924318

[47] Shinkai H: Cholesteryl ester transfer protein inhibitors and their potential for the treatment of cardiovascular diseases. *Vasc Health Risk Manag.* 2012;8:323–31 10.2147/VHRM.S25238 22661899 PMC3363149

[48] Miyares MA Davis K: Patient considerations and clinical impact of cholesteryl ester transfer protein inhibitors in the management of dydlipidaemia: focus on anacetrapib. *Vasc Health Risk Manag.* 2012;8:483–93 10.2147/VHRM.S29010 22977305 PMC3430392

[49] deGoma EM Rader DJ: High-density lipoprotein particle number: a better measure to quantify high-density lipoprotein? *J Am Coll Cardiol.* 2012;60(8):517–20 10.1016/j.jacc.2012.03.058 22796252

[50] Kingwell BA Chapman MJ Kontush A: HDL-targeted therapies: progress, failures and future. *Nat Rev Drug Discov.* 2014;13(6):445–64 10.1038/nrd4279 24854407

[51] A study of evacetrapib in high risk vascular disease. Reference Source

[52] Nicholls SJ Brewer HB Kastelein JJ: Effects of the CETP inhibitor evacetrapib administered as monotherapy or in combination with statins on HDL and LDL cholesterol: a randomized controlled trial. *JAMA.* 2011;306(19):2099–109 10.1001/jama.2011.1649 22089718

[53] Randomized evaluation of the effects of anacetrapib through lipid-modification. Reference Source

[54] Cannon CP Shah S Dansky HM: Safety of anacetrapib in patients with or at high risk for coronary heart disease. *N Engl J Med.* 2010;363(25):2406–15 10.1056/NEJMoa1009744 21082868

[55] Karlberg JPE Speers MA: Reviewing clinical trials: A guide for ethics committees.2010 Reference Source

