# Supplementary material for: CETP inhibitors and cardiovascular disease: Time to think again
Source: F1000Res. 2014 Jun 10;3:124. [Version 1] doi: 10.12688/f1000research.4396.1 (PMC4149245; doi:10.12688/f1000research.4396.1)
Supplement: Peer review history

# Peer review: Referee response for version 1

**DOI:** 10.5256/f1000research.4705.r5614

**Published:** 2014-07-29

**Authors:** Cesare Sirtori

**Affiliations:** 1 Department of Pharmacological Sciences, University of Milan, Milan, Italy

This paper by Miller attempts to elucidate once and for all the present role of CETP and CETP antagonists in coronary prevention. The message is clear and well written. I do have a question on the structure of the paper however: while the background of CETP from the earlier papers by Havel is clear and well written, I question the idea of separating this from the very informative Table 1 which collates two separate pieces of information and is otherwise unclear.

Also, as the current trend is to evaluate genomic analysis, in particular by Mendelian randomization, this could indicate that HDL is not a genetic risk component. This should be given special emphasis, since the Mendelian randomization analysis was focused on LIPC (just one determinant of HDL levels) whereas the author points out that SNPs of CETP are also probably involved. However he gives no explanation of the mechanism of these SNPs, which are apparently associated with raised HDL and also with reduced incidence of MI.

In my view, the major focus of this paper should be on drugs, especially as drugs are still used to antagonize CETP in clinical trials. In the studies on torcetrapib it was noted ( Nicholls *et al,Circulation* 2008) that individuals with the highest post-treatment HDL-cholesterol are apparently protected from coronary disease. Does this make sense? After all if HDL is traveling through the blood in very large amounts without going back to the artery this could provide some potential protection, although this should be evaluated with more rigor. A final note on the studies with torcetrapib is the incremental elevation of blood pressure. The rise of blood pressure was of such little entity (at most 5 mmHg) that just posting these data in a risk score (Framingham or other) allowed one to conclude that this influences risk minimally and does  modify the enormous change in risk consequent to the HDL rise elicited by the CETP inhibitor ( Sirtori, Mombelli, *Clin. Chem,* 2010).

In regards to the rabbit studies: this started with the Okamoto paper in Nature ( 2000), which had one serious problem; the control group only had a final cholesterol of 129 mg/dl. Thus, in my view, the apparent advantage of giving JT -705 (dalcetrapib) makes no sense. A much better conducted study published by Huang ( Huang *et al,Clin Sci,* 2002) used  a similar protocol but had a cholesterol of 757 mg/dl in the control group. In this study, in spite of a marked rise of HDL-C, there was no arterial benefit. This, in my view, should have closed the story. Unfortunately it did not.

The probucol data, on the other hand, are definitely of high significance. Probucol raises CETP and prevents arterial disease (a number of reports have recently come from Japanese investigators e.g. Kasai *et al.Atherosclerosis.* 2012). Most excitingly, it removes cholesterol deposits (xanthelasmas/xanthomas) thus indicating that increased CETP is beneficial in man. Another negative issue to be raised is that in the Brusseau paper ( ref 40) there was no evidence of a reduced cholesterol pool/increased fecal steroid excretion following torcetrapib, indicating again that blocking CETP does not in any way improve cholesterol turnover. I tend to believe that the issue of apo A-I syntheis is not of major significance. The reduction of AI synthesis (probucol) and increased synthesis (torcetrapib) is therefore of little interest.

In conclusion the author has certainly done a very good job but the paper would be improved by the inclusion of some older data, that in my view are more significant, vs data provided by the recent overviews or Mendelian randomizations.

I have read this submission. I believe that I have an appropriate level of expertise to confirm that it is of an acceptable scientific standard.

# Peer review: Referee response for version 1

**DOI:** 10.5256/f1000research.4705.r5462

**Published:** 2014-07-17

**Authors:** Gerald Watts

**Affiliations:** 1 School of Medicine and Pharmacology, University of Western Australia, Crawley, WA, Australia

This is an exceptionally good review and balanced assessment of the status of CETP inhibitors and ASCVD from a world authority in the field. The article highlights important data that might have been overlooked when promulgating the clinical value of CETPIs and related trials.

Only 2 areas need revision:

1. Page 3, para 2: the notion that these data from Papp *et al.* convey is critical and the message needs an explicit sentence or two at end of paragraph.
2. Page 4, Conclusion: the assertion concerning the ethics of the two Phase 3 clinical trials needs toning down. Perhaps rephrase to indicate that the value and sense of doing these trials is open to question, with attendant ethical implications, or softer wording to that effect.

I have read this submission. I believe that I have an appropriate level of expertise to confirm that it is of an acceptable scientific standard.
